# Chemotherapy-induced peripheral neuropathy: part 1—current state of knowledge and perspectives for pharmacotherapy

**DOI:** 10.1007/s43440-020-00109-y

**Published:** 2020-05-11

**Authors:** Kinga Sałat

**Affiliations:** Department of Pharmacodynamics, Chair of Pharmacodynamics, Jagiellonian University Medical College, 9 Medyczna St., 30-688 Krakow, Poland

**Keywords:** Chemotherapy-induced peripheral neuropathy, Neuronal hyperexcitability, Platinum derivatives, Paclitaxel, Vinca alkaloids, Bortezomib

## Abstract

**Background:**

Despite the increasing knowledge of the etiology of neuropathic pain, this type of chronic pain is resistant to available analgesics in approximately 50% of patients and therefore is continuously a subject of considerable interest for physiologists, neurologists, medicinal chemists, pharmacologists and others searching for more effective treatment options for this debilitating condition.

**Materials and methods:**

The present review article is the first of the two articles focused on chemotherapy-induced peripheral neuropathy (CIPN).

**Results:**

CIPN is regarded as one of the most common drug-induced neuropathies and is highly pharmacoresistant. The lack of efficacious pharmacological methods for treating CIPN and preventing its development makes CIPN-related neuropathic pain a serious therapeutic gap in current medicine and pharmacotherapy. In this paper, the most recent advances in the field of studies on CIPN caused by platinum compounds (namely oxaliplatin and cisplatin), taxanes, vinca alkaloids and bortezomib are summarized.

**Conclusions:**

The prevalence of CIPN, potential causes, risk factors, symptoms and molecular mechanisms underlying this pharmacoresistant condition are discussed.

**Graphic abstract:**

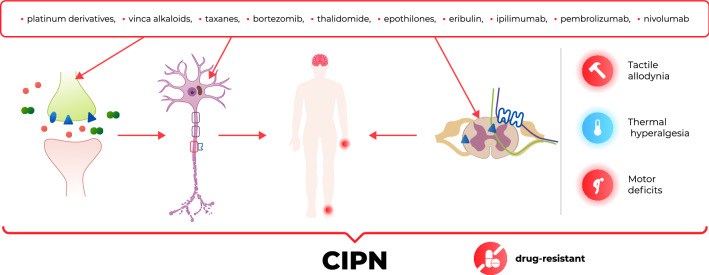

## Introduction

### Neuropathic pain as a major feature of neuropathy – General characteristics: Definition, epidemiology, symptoms and risk factors

According to the International Association for the Study of Pain’s 2011 definition, neuropathic pain is pain that arises as a direct consequence of a lesion or disease affecting the somatosensory system. This definition emphasizes the involvement and a key role of the somatosensory system in this clinical entity to distinguish it from other types of pain that may also be associated with disorders of the motor system [1].

Neuropathic pain is a chronic neurological disorder that represents a broad category of pain syndromes that include various peripheral and central nervous system impairments called neuropathies [2]. As a disease itself, neuropathic pain has a significantly negative impact on the sleep, everyday functioning and quality of life of patients. Therefore, it is also regarded as one of the triggering factors of anxiety and depression symptoms within the patient population [1].

A precise estimation of the incidence of neuropathic pain is difficult because of the lack of simple diagnostic criteria for large epidemiological surveys of the general population [1]. Epidemiological data estimate that the overall prevalence of neuropathic pain may be as high as 7–8%, which corresponds to 20–25% of individuals with chronic pain. The values reported here may vary significantly for particular types of neuropathic pain, such as postherpetic neuralgia (8–10%), painful diabetic neuropathy (14–26%), and neuropathic pain related to surgery (10–50%), multiple sclerosis (20–30%), spinal cord injury (30–40%) and cancer (17–19%) [3].

Clinically, neuropathic pain is characterized by so-called positive and negative symptoms. Positive symptoms include various painful symptoms (tactile and thermal allodynia or hyperalgesia and spontaneous pain episodes), paresthesias, and dysesthesia (e.g., tingling, prickling, burning, knifelike, electrical, pulling or tightening sensations). Negative symptoms usually include neurological sensory deficits (numbness and the feeling of wearing socks all the time) in the painful area and are frequently accompanied by other deficits, such as motor or cognitive impairments [3–5].

Neuropathic pain is regarded as a heterogeneous and multidimensional clinical entity. For example, peripheral neuropathic pain comprises postherpetic neuralgia and traumatic nerve injury, but it should be noted that many of these patients may suffer from mixed pain syndromes involving both neuropathic and nonneuropathic components (e.g., lumbar or cervical radiculopathies). Central neuropathic pain syndromes are also common, being observed in up to 8% of stroke patients, 30–50% of patients with a spinal cord injury and up to 20–25% of patients with multiple sclerosis or Parkinson’s disease. Thus, neuropathic pain patients with distinct sensory profiles may respond differently to available treatment options.

**Fig. 1:**
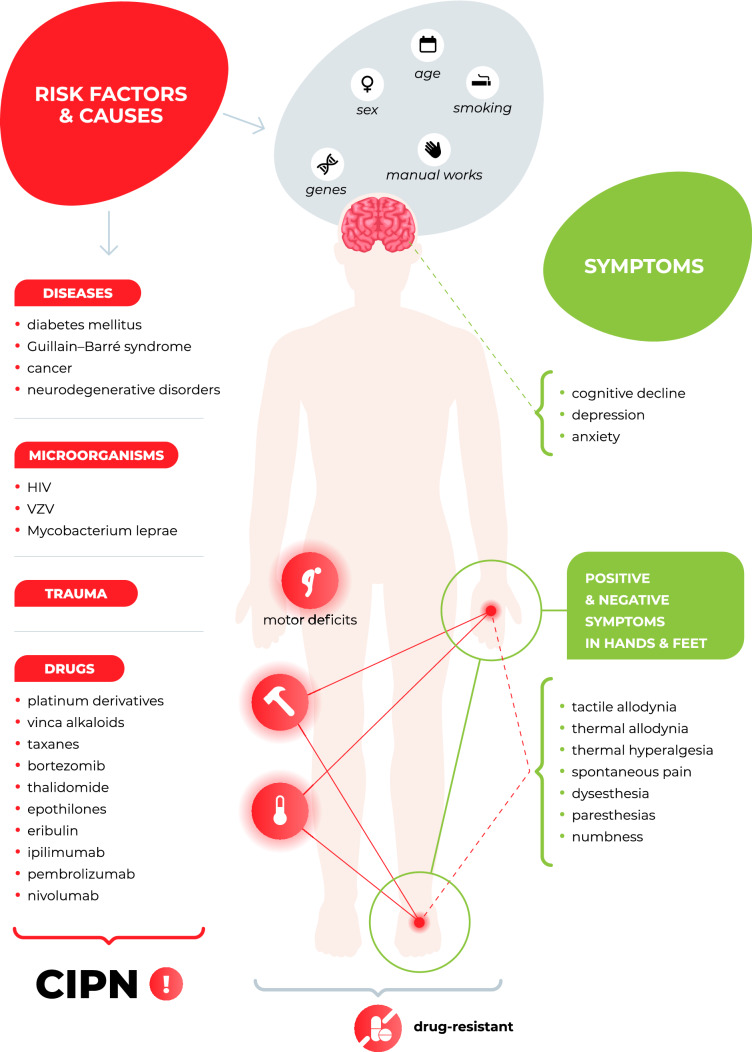
Neuropathy—Causes, risk factors and symptoms

In recent years, several risk factors for neuropathy and neuropathic pain, including age > 60 years, female sex, participation in manual labor [3, 6, 7], and smoking history [8, 9] have been identified, and the number of potential causes of neuropathic pain is still increasing (Fig. 1). These causes include metabolic (diabetic), infectious (varicella zoster and human immunodeficiency virus infections and *Mycobacterium leprae*), traumatic, immune (Guillain–Barre syndrome), inherited (e.g., inherited erythromelalgia caused by mutations in SCN9A, which encodes the voltage-gated sodium channel Na_v_1.7) and drug-induced factors [1].

Despite the increasing knowledge of the etiology of neuropathic pain, this type of chronic pain is resistant to available analgesics in approximately 50% of patients [2, 10] and, therefore, is continuously a subject of considerable interest for physiologists, neurologists, medicinal chemists, pharmacologists and others searching for more effective treatment options for this debilitating condition.

The present review article is the first of two articles focused on chemotherapy-induced peripheral neuropathy (CIPN), which is one of the most common drug-induced neuropathies and is highly pharmacoresistant.

## CIPN—Definition and causes

Epidemiological data presented by the World Health Organization indicate that cancer is the second leading cause of death worldwide following heart disease. It was responsible for approximately 9.6 million deaths in 2018, and the high mortality rate of cancer makes it a civilizational disease often termed as ‘a cancer epidemic’ [11, 12].

In the last decades, innovative systemic anticancer therapies have been used more broadly [13]. This, together with progress in the early detection of tumors and the high success rate of cancer treatment, has increased the number of cancer survivors. On the other hand, with the growing use of antitumor drugs, the prevalence of serious adverse iatrogenic effects caused by antitumor drugs have become a significant clinical issue in the cancer patient population [14].

**Fig. 2:**
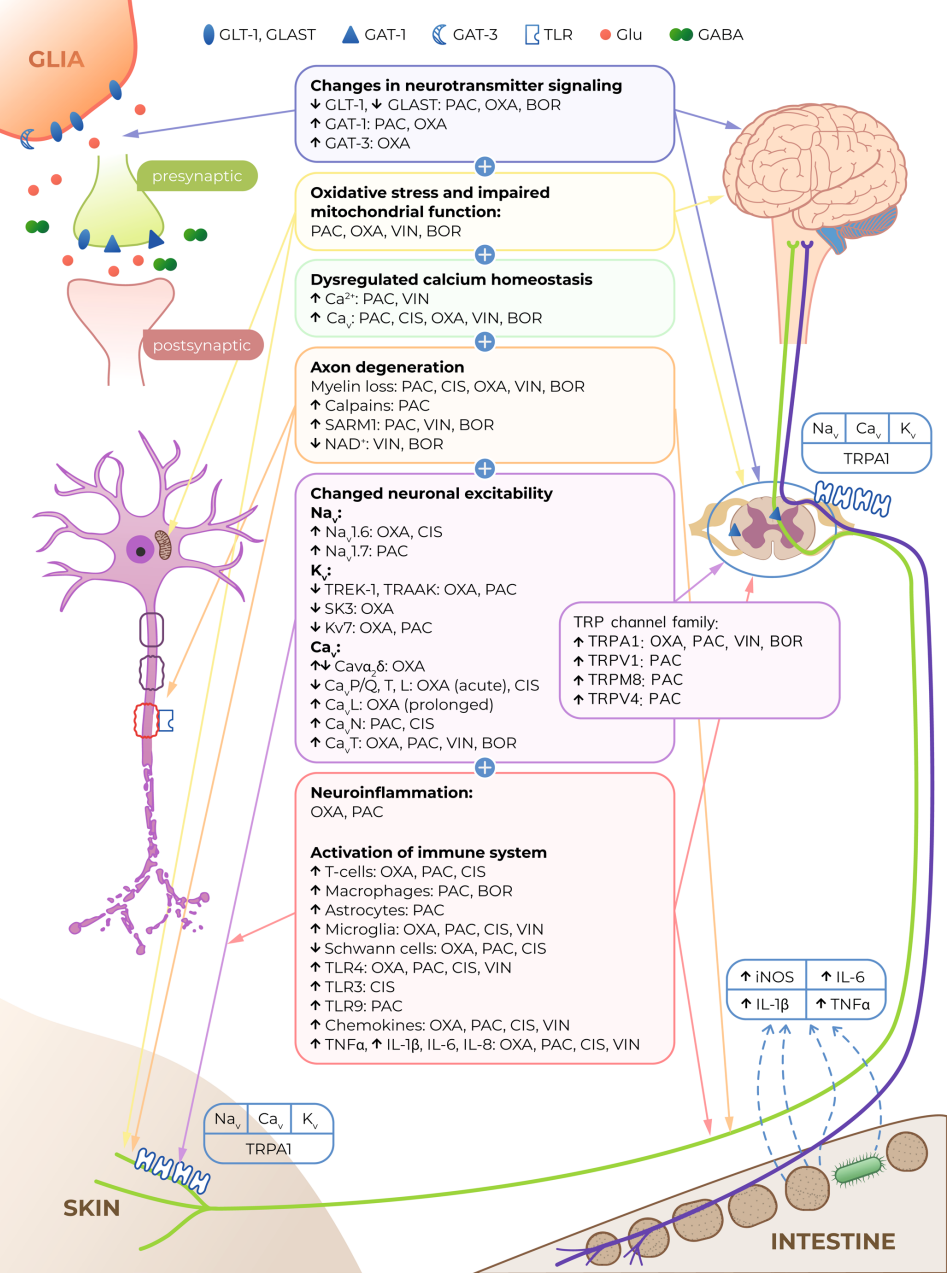
Antitumor drugs that induce CIPN and the main mechanisms underlying CIPN [18–21]. *GLT-1 *glutamate transporter 1, *GLAST* GLutamate and ASpartate Transporter, *GABA* γ-aminobutyric acid, *GAT* GABA transporter, *TLR* toll-like receptor, *Glu* glutamate, *PAC* paclitaxel, *VIN* vincristine, *OXA* oxaliplatin, *CIS* cisplatin, *BOR* bortezomib, *Na*_*v*_ voltage-gated sodium channels, *Ca*_*v*_ voltage-gated calcium channels, *K*_*v*_ voltage-gated potassium channels, *TRPA1* Transient Receptor Potential Ankyrin-repeat 1 channel, *TRPV* Transient Receptor Potential Vanilloid channel, *TRPM8* Transient Receptor Potential Melastatin 8 channel, *iNOS* inducible nitric oxide synthase, *IL* interleukin, *TNFα* tumor necrosis factor α, *SARM1* sterile alpha and TIR motif-containing protein 1, *NAD +* nicotinamide adenine dinucleotide

CIPN is one of the most common dose-limiting adverse effects of many chemotherapeutic agents, such as platinum derivatives, taxanes, vinca alkaloids, thalidomide, epothilones, eribulin, ipilimumab, pembrolizumab, nivolumab and bortezomib (Figs. 1, 2). It affects > 60% of patients receiving anticancer therapy. Although CIPN is a nonfatal condition, it significantly worsens patients’ quality of life and is usually regarded as very troublesome, as it might not only influence the efficacy of antitumor agents but also be a major cause of ongoing pain in cancer survivors [15–17].

At present, CIPN is often considered an unavoidable adverse effect of cancer chemotherapy that should be accepted by cancer patients and clinicians in the light of the extended life-span offered by these drugs. Since the major manifestation of CIPN comprises severe pain episodes involving tactile and thermal allodynia, hyperalgesia and spontaneous pain, analgesic drugs are used in patients exposed to CIPN-inducing antitumor therapy. However, it should be noted that the analgesic drugs that effectively relieve pain symptoms in CIPN and are used as interventional treatments for pre-existing CIPN-related pain are very limited and that their efficacy in CIPN is significantly lower than that observed in other neuropathic pain types. Importantly, there are currently no recommended options for effectively preventing neuropathic pain in CIPN [19], and strong evidence for the utility and clinical efficacy of some previously tested preventive therapies (e.g., pregabalin, gabapentin, duloxetine, calcium/magnesium infusion, amifostine, glutathione, glutamine, acetyl-l-carnitine and erythropoietin) is still limited [22]. The lack of efficacious pharmacological methods for treating CIPN and preventing its development [23] makes CIPN-related neuropathic pain a serious therapeutic gap in current medicine and pharmacotherapy. So far, there has been only one potential drug candidate for preventing the development of oxaliplatin-induced acute and delayed CIPN, namely, calmangafodipir, a mitochondrial manganese superoxide dismutase mimetic, which is currently being studied in a placebo-controlled, double-blinded randomized phase III study [24]. Therefore, basic science research in this area and large clinical trials are urgently needed to establish novel and effective therapeutic solutions to prevent this devastating condition [17]. There seems to be a strong demand for a more thorough understanding of the etiology of CIPN, which would help to develop effective mechanism-based disease-modifying therapies. Importantly, such approaches should not negatively influence the antitumor effects of the chemotherapeutics used [19, 23].

Only few studies have been conducted to compare directly the characteristics of CIPN and other neuropathies. As mentioned above, these studies have shown that neuropathic pain in the course of CIPN is more pharmacoresistant than other neuropathic pain types but, on the other hand, some common mechanistic features have also been shown. Importantly, in a manner similar to other peripheral neuropathies, in CIPN the central nervous system is affected due to the changes in the barrage of peripheral input (discussed in “Central nervous system structures and neurotransmitters”). Therefore, many analgesic drugs used for alleviating CIPN-related neuropathic pain are also used in neuropathic pain of other origin.

A direct comparison between diabetic neuropathy and CIPN has been conducted by Jin and colleagues [25] with respect to symptom severity and therapeutic responses. Using a rat model, they compared peripheral nerve damage due to hyperglycemia (i.e., painful diabetic neuropathy) with that caused by paclitaxel treatment. Biochemical, sensory and immunohistochemical parameters of cutaneous and sciatic nerves and the therapeutic effects of test drugs (alpha-lipoic acid and DA-9801) were compared in these two models. Sensory thresholds of animals to mechanical, heat, and pressure stimuli were altered by both hyperglycemia and paclitaxel when compared with controls. There were no significant differences in the biochemical markers of blood glutathione between diabetic rats and the paclitaxel-treated group. Quantitative comparisons of peripheral nerves by intraepidermal nerve fiber density analysis indicated that both groups were similar, but nerve density was significantly improved after alpha-lipoic acid and DA-9801 treatment in diabetic animals but not in the paclitaxel-treated groups. Sciatic nerves were less damaged in the paclitaxel-treated groups compared with the diabetic group. Hence, it was concluded that the manifestation of neuropathy, as well as some therapeutic responses in CIPN may be different from those observed in other peripheral neuropathies.

Similarity between paclitaxel-induced CIPN model and short-term models of traumatic neuropathy has also been demonstrated [26].

### Prevalence of CIPN and risk factors

As mentioned above, the survival rates of patients treated with antitumor agents are increasing. Hence, CIPN and CIPN-related neuropathic pain episodes have become a significant clinical issue among cancer survivors [15]. In general, the prevalence of CIPN resulting from different antitumor drugs and doses varies significantly, with reported prevalence rates ranging from 19 to more than 85% [17]. It is estimated that approximately 70% of patients receiving chemotherapy develop CIPN during the first month of treatment [17, 20], whereas in approximately 20–30% of these patients, CIPN might be converted to a chronic, persistent and highly pharmacoresistant form [17, 23] that can be observed 6 months or even longer after therapy cessation [14, 16, 27, 28]. Importantly, the symptoms of these delayed complications may persist for several months and can be progressively aggravated; this phenomenon in which either mild neuropathy worsens or a new form of CIPN develops is termed ‘coasting’ [21]. Since chemotherapeutics are not being applied when this disorder develops, coasting is a great challenge for clinicians; patients may be cancer-free but suffer from neuropathy evoked by previously received anticancer treatment [17].

Importantly, CIPN tends to occur both in adults and in younger patients. In children, most cases of CIPN are due to the use of vincristine and platinum derivatives, as other CIPN-inducing chemotherapeutics are not routinely used in this population [15]. It should also be noted that the prevalence and symptoms of CIPN seem to be age-related. This is thought to result from the distinct neurobiology of the peripheral nervous system in children and adults. The diameter, density and myelination of axons in the dorsal root ganglion significantly change during childhood to achieve full maturation, and this may have a strong influence on the risk of development and severity of CIPN. This notion is supported by previous observations that vincristine causes motor deficits more frequently in pediatric patients than adults. The reason for this seems to be unclear, and several potential mechanisms, including the use of higher doses, altered pharmacokinetics, different neuronal biology, and the lack of early detection of neurotoxic symptoms, should be considered [15]. The treatment of CIPN symptoms, recovery, and the delayed effects of chemotherapy may also vary between adult and pediatric populations. Importantly, drugs commonly used to treat neuropathic pain in adult patients (e.g., duloxetine) have not been widely studied in children. Also, physical and exercise-based therapies have not been evaluated in children receiving CIPN-inducing drugs. Thus, both the symptoms of CIPN and methods for alleviating them might be different between these two populations [15].

In addition to age, a number of other potential risk factors for CIPN development have been identified. These include the cumulative dose of a chemotherapeutic agent, genetic factors, a history of neuropathy before the start of chemotherapy (e.g., painful diabetic neuropathy or neuropathy due to viral infections), impaired renal function with reduced creatinine clearance, and smoking history [9, 15, 17, 28–32].

### Symptoms of CIPN

Within the patient population, numerous symptoms of CIPN have been identified, all of which seriously worsening patients’ quality of life [14]. It has been widely reported that patients who develop CIPN symptoms have significant difficulties in essential daily functioning. In these patients, unsteady gait (numbness and the loss of joint position sense), difficulties in fine finger movements, pain during walking (mechanical allodynia), and cold-exacerbated pain episodes (cold hypersensitivity) have been reported [17].

In general, the characteristics, time of onset and duration of CIPN symptoms depend on the chemotherapeutic agent used. Most frequently these symptoms appear after repeated (three to four) cycles of therapy; however, the immediate manifestation of CIPN symptoms has also been noted and in some patients, these symptoms might become permanent and continue for years (‘coasting’) [33, 34].

CIPN is a disease with a plethora of symptoms associated with distress (mood disorders: anxiety, depression [35], sleep disorders [36], cognitive impairments (chemobrain, a.k.a. chemofog [37, 38]), fatigue, altered taste sensation, and nausea), and a wide range of neurological symptoms present mainly in the hands and feet (e.g., numbness, tingling, pain, muscle weakness and sensitivity to cold or heat) are also observed [14, 29, 39]. These symptoms can be classified as a motor or sensory impairments. These impairments typically include sensory axonal peripheral neuropathy (‘stocking and glove’ neuropathy), which is accompanied by numbness, paresthesias, ongoing/spontaneous pain hypersensitivity to mechanical and thermal stimuli in the hands and feet [17], and motor symptoms such as distal weakness, reduced or absent Achilles tendon reflexes, paresthesias, breath dysregulation, abnormal swallowing, laryngospasm, muscle cramps, jaw stiffness, visual field changes, muscle weakness, reduced balance control and insecure gait, the mechanisms of which are not fully understood [20, 33, 40, 41].

### Molecular basis of CIPN

The mechanisms of action through which antitumor drugs induce cell death and inhibit cell proliferation are diverse but very well defined. It is, however, not entirely clear, if these effects are also responsible for the damage to nonproliferating sensory neurons that underlies CIPN development. Available data from basic science studies indicate that in addition to direct cytotoxic effects, other cell-type-specific actions resulting from the on-target pharmacological effects of CIPN-inducing antitumor drugs might also contribute to the development of CIPN and decreased viability of sensory neurons. In addition, off-target effects may also be implicated in CIPN development, but this issue requires further and more detailed research [20]. Therefore, CIPN is regarded a multifactorial disorder with diverse pharmacological mechanisms, and a number of common pathologies, including altered ion channel functions, oxidative stress, axonal degeneration, impaired calcium homeostasis, neuroinflammation, and activation of the immune system, have been proposed to underlie CIPN development (Fig. 2).

#### Oxidative stress

In addition to controlling cellular energy production and energy supply, mitochondria are also involved in the regulation of cell death. Neuronal mitochondrial dysfunction resulting in nitro-oxidative stress [42] is regarded as one of the most important contributors to CIPN development [23, 29, 43].

Several antitumor drugs, including oxaliplatin, cause damage to neuronal and nonneuronal mitochondria, thus leading to oxidative stress mediated by redox-sensitive TRPA1 channels. These are biomolecules that are also implicated in oxaliplatin-induced mechanical and cold hypersensitivity [44–50]. Oxidative stress increases the production of proinflammatory mediators, which further destroy mitochondria, enhance the production of reactive oxygen species and contribute to various pathological processes resulting from oxidative stress. These phenomena might also cause demyelination and disruption of the cytoskeleton of peripheral nerves [51].

Oxaliplatin has been shown to bind to mitochondrial DNA and form adducts that cannot be repaired due to the lack of DNA repair systems within mitochondria. These adducts impair mitochondrial DNA replication and transcription, leading to altered protein synthesis and impaired respiratory chain function. In addition, vincristine dysregulates neuronal mitochondria, which leads to altered neuronal excitability and glial cell dysfunction. The influence of vincristine on mitochondria is thought to involve altered mitochondrial calcium signaling, and the maintenance of intracellular calcium homeostasis is beneficial for preventing CIPN [51, 52]. Paclitaxel does not directly affect mitochondrial DNA but induces swollen and vacuolated mitochondria in both myelinated and unmyelinated sensory axons, and these changes are accompanied by increased production of reactive oxygen species in the nervous system [20, 53–55].

The effect of the CIPN-inducing proteasome inhibitor bortezomib on mitochondria in sensory neurons has also been investigated [56]. In a previous study bortezomib was shown to alter the metabolic phenotype of sensory neurons and increase the production of metabolites due to aerobic glycolysis, thus resulting in CIPN development. It was also demonstrated that the targeted inhibition of the enzymes that maintain aerobic glycolysis might be a strategy for treating CIPN.

#### Dysregulated calcium homeostasis

Impaired calcium homeostasis and calcium signaling have also been shown to contribute to the development of oxaliplatin-, cisplatin-, vincristine-, and paclitaxel-induced CIPN. This calcium-dependent neurotoxic pathway is particularly important for oxaliplatin, of which oxalate, a well-known calcium chelator that contributes to the development of oxaliplatin-induced acute form of CIPN, is a metabolite.

Unlike that of oxaliplatin-induced neuropathy, the contribution of calcium ions to cisplatin-induced neuropathy is relatively poorly understood. Cisplatin increases the expression of N-type voltage-gated calcium channels (Ca_v_2.2) [57, 58], but the role of these channels in the clinical manifestation of cisplatin-induced CIPN remains unclear. Calcium signaling has also been reported to participate in CIPN induced by paclitaxel [55], which causes rapid mitochondrial depolarization and calcium release in both neuronal and nonneuronal cells, possibly via the activation of the mitochondrial permeability transition pore [55, 59].

Increased expression of low-voltage-activated calcium channels (T-type channels; Ca_v_3.2) has also been shown in paclitaxel-exposed rat dorsal root ganglia. T-type current amplitudes are increased 7 days after paclitaxel treatment [60].

Maintaining intracellular calcium homeostasis attenuates CIPN caused by vincristine [52].

Increased protein levels of Ca_v_3.2 in primary afferents caused by decreased proteasomal degradation of Ca_v_3.2 (T-type) channels is involved in bortezomib-induced neuropathic pain [61].

#### Axonal degeneration

Animal and human studies have demonstrated that the long-term use of the platinum derivatives [62, 63] paclitaxel [64], vincristine [65], and bortezomib [66] can induce axonal degeneration, specifically, the loss of large myelinated, small unmyelinated and intra-epidermal nerve fibers. The loss of myelin and changes to the axonal cytoskeleton may alter the structure and impair the function of peripheral nerves, thus leading to the development of sensory and motor peripheral neuropathy and altered pain perception. The molecular mechanisms of these phenomena are not fully established [43, 63, 67].

In patients, oxaliplatin causes moderate axonal degeneration and the loss of intra-epidermal nerve fibers, while cisplatin induces axonal degeneration of large myelinated fibers and myelin disruption. Similar pathologies have also been noted within myelinated fibers of the sciatic nerve in cisplatin-treated mice [33, 68].

The degeneration of distal sensory axons, demyelination and nerve fiber loss have also been observed in vincristine-, bortezomib-, and paclitaxel-induced neuropathy [20, 69].

A zebrafish model has been used to study the mechanisms underlying paclitaxel-induced neurotoxicity, sensory axon degeneration and the loss of touch response in the distal caudal fin. In zebrafish larvae, paclitaxel induces epithelial damage and reduces the mechanical stress resistance of the skin prior to the induction of axonal degeneration. Reduced healing capacity of the injured skin of paclitaxel-treated zebrafish and scratch-wounded HEK001 human keratinocytes has also been demonstrated. The results of this research demonstrated that paclitaxel induces the upregulation of matrix-metalloproteinase 13 in the skin and promotes epithelial damage that precedes axonal degeneration [70].

In CIPN, calpains, as calcium-dependent proteases, have been shown to contribute to axonal degeneration [69]. Paclitaxel induces CIPN by altering inositol trisphosphate (IP_3_) receptor phosphorylation and intracellular calcium flux. It also activates calpain proteases [71].

Recent studies have also identified a new molecular mechanism involving sterile alpha and TIR motif-containing protein 1 (SARM1) as an injury-inducible NADase that triggers axonal loss [9, 67]. The activation of SARM1 has been shown to contribute to Wallerian degeneration, a phenomenon that occurs during axonal degeneration [72]. The genetic deletion of SARM1 protects axons from degeneration after axotomy in mice. Turkiew and colleagues showed that paclitaxel-induced CIPN model mice lacking the Sarm1 gene are resistant to distal axonal degeneration [64].

Subacute/chronic axonal loss induced by vincristine also involves the SARM1-mediated axonal destruction pathway, and the genetic deletion of SARM1 blocks the development of bortezomib- and vincristine-induced CIPN in mice [65, 72].

The induction of CIPN by axonal degeneration is thought to be due to a loss of nicotinamide adenine dinucleotide (NAD +) via the SARM1 pathway [73]. Using cultured neurons, Geisler and colleagues found that vincristine and bortezomib are able to induce an axonal degeneration program that involves the nicotinamide mononucleotide NMNAT2, SARM1, and the loss of NAD + . Both drugs activate SARM1 and cause the SARM1-dependent depletion of axonal NAD + , which is followed by local metabolic collapse, axonal fragmentation and axonal degeneration. Of note, the actions of vincristine and bortezomib involve different mechanisms, i.e., Wallerian degeneration occurs after vincristine administration, while bortezomib induces apoptosis [72].

It has also been suggested that vincristine might be responsible for an excessive increase in a NAD + direct precursor, i.e., a nicotinamide mononucleotide, rather than a decreased level of NAD + and that this is also likely to play a role in CIPN development [73].

#### Changes in neuronal excitability

Altered peripheral nerve excitability has also been implicated in CIPN development. The altered expression and functional impairment of voltage-gated sodium (Na_v_), voltage-gated potassium (K_v_), voltage-gated calcium (Ca_v_) and transient receptor potential family (TRP) channels are regarded as the most prominent phenomena underlying CIPN caused by the platinum derivatives vincristine, paclitaxel and bortezomib [17, 74].

##### Na_v_ channels

Na_v_ 1.6 channels appear to be involved in the development of oxaliplatin-induced cold hyperalgesia [75], and the acute oxaliplatin-induced aggravation of cold hypersensitivity is abolished by the administration of a selective Na_v_1.6 inhibitor [76]. Our recent study also demonstrated that ambroxol, a Na_v_ channel inhibitor, alone or in combination with pregabalin efficiently attenuates cold allodynia in oxaliplatin-treated mice; this suggested that Na_v_ channels participate in this phenomenon [77]. The acute neurotoxicity caused by oxaliplatin is thought to be due to the accumulation of its metabolite oxalate, which is a calcium chelator. It immobilizes calcium ions and alters calcium-sensitive Na_v_ channel kinetics [78].

Clinically, the role of Na_v_ channels in CIPN development was also confirmed in a cisplatin-treated patient in whom lacosamide, an antagonist of Na_v_ channels, significantly alleviated the severe painful symptoms of CIPN [79]. However, it should be noted that in addition to Na_v_ channels, other mechanisms have been implicated in cisplatin- and carboplatin-induced CIPN. Avan and colleagues demonstrated that both these drugs are neurotoxic because they induce DNA adduct accumulation and the inhibition of DNA repair pathways, such as the extracellular signal-regulated kinase 1/2, c-Jun N-terminal kinase/stress-activated protein kinase, and p38 mitogen-activated protein kinase pathways, which ultimately results in apoptosis [78].

The gene expression and currents of Na_v_ 1.7 channel (Na_v_1.7) are increased in the dorsal root ganglia of paclitaxel-treated rats. Similarly, gain-of-function mutations in human Na_v_1.7 channels have also been noted in paclitaxel-treated patients [80, 81].

##### K_v_ channels

In isolated rat sciatic nerves, oxaliplatin causes broadening of the repolarization phase, induced repetitive firing and afterhyperpolarization, and these effects are caused by its effect on K_v_ channels. After oxaliplatin and paclitaxel treatment, decreased expression of two-pore domain K^+^ channels (TREK-1 and TRAAK) is observed in the rodent dorsal root ganglia [20]. In mice, riluzole, an activator of TREK-1 and TRAAK, prevents both sensory and motor deficits induced by oxaliplatin and attenuates the depression-like symptoms induced by oxaliplatin. Riluzole has no negative effect on the antiproliferative capacity of oxaliplatin in human colorectal cancer cells and does not reduce its anticancer effect in a mouse model of colorectal cancer. Moreover, riluzole decreases the viability of a human colorectal cancer cell line in vitro and inhibits polyp development in vivo. These data confirm the important role of TREK-1 and TRAAK in CIPN [82, 83].

A potential role for the potassium channel SK3 (KCNN3 gene) in oxaliplatin-induced CIPN has also been suggested. In a preclinical model, a slight association between CIPN and CAG repeat polymorphisms in the KCNN3 gene was shown, but such an association between CIPN and KCNN3 polymorphisms was not shown in patients [84]. In a mouse study, the activation of K_v_7 channels reduced the symptoms of CIPN induced by oxaliplatin and paclitaxel [85]. In line with this, minoxidil, a potassium channel opener alleviates the signs of paclitaxel-related CIPN [17].

##### Ca_v_ channels

Increased expression of Ca_v_*α*_2_*δ *– 1 subunit mRNA and protein in the spinal cord 2 and 4 days after oxaliplatin treatment contributes to acute cold hypersensitivity in rodents [86]. A crucial role of the Ca_v_*α*_2_*δ *− 1 subunit in the development of oxaliplatin-induced acute and delayed cold hypersensitivity has been shown in mice [77] and rats [86].

The short- and long-term effects of the exposure of Ca_v_ channels to oxaliplatin have been investigated in rat small dorsal root ganglia. Ca_v_ channel currents are concentration-dependently reduced by oxaliplatin. Moreover, differential time-dependent effects of oxaliplatin on calcium currents have been noted. Acute treatment with oxaliplatin leads to a reduction in P/Q-, T-, and L-type Ca_v_ channel currents, while there is no effect of this acute treatment on N-type Ca_v_ channel currents. In contrast to this, prolonged (24 h) exposure of dorsal root ganglia neurons to oxaliplatin significantly increases L- and T-type Ca_v_ channel currents. Increased L- and T-type Ca_v_ channel protein levels in dorsal root ganglion neurons have also been noted 24 h after oxaliplatin exposure [87]. Analogous research showing a crucial role of N-type Ca_v_ channels in cisplatin-induced polyneuropathy was conducted. This study showed that cisplatin reduced Ca_v_ channel currents in a concentration-dependent manner. Subtype-specific measurements of Ca_v_ channel currents showed the differential effects of this platinum derivative on Ca_v_ channel subtypes. While the currents of P/Q-, L- and T-type Ca_v_ channels were reduced, those of N-type Ca_v_ channels were increased. Further analyses revealed an increase in N-type Ca_v_ channel protein levels in dorsal root ganglion neurons 24 h after cisplatin administration. Cisplatin activated caspase-3 and this effect were prevented by *ω*-conotoxin MVIIA, an N-type Ca_v_ channel inhibitor [58].

A recent study [88] showed that cisplatin-induced neurotoxicity might be due to functional alterations in Ca_v_ channels but not to structural damage to these channels, as no morphological signs of damage, apoptosis or necrosis were noted in dorsal root ganglion cells exposed to cisplatin for 26 days.

Increased levels of Ca_v_ channel mRNA within dorsal root ganglion neurons have been demonstrated in paclitaxel-treated mice, and Ca_v_ channel antagonists (gabapentin and ethosuximide) effectively reduce hyperalgesia in rodents with paclitaxel- and vincristine-induced CIPN [17]. Many studies have confirmed a crucial role of N-type and T-type Ca_v_ channels in paclitaxel-induced CIPN [74] as well as a key role of T-type Ca_v_ channels in CIPN caused by vincristine [89].

A mouse study showed that the inhibition of the proteasomal degradation of T-type Ca_v_ channels by bortezomib and bortezomib-induced increases in the levels of USP5, a deubiquitinating enzyme that specifically inhibits the proteasomal degradation of T-type Ca_v_ channels, increases the protein levels of T-type Ca_v_ channels in nociceptors. The systemic administration of T-type calcium channel blockers reverses CIPN caused by bortezomib. Also in ND7/23 cells, bortezomib increases the protein levels of T-type Ca_v_ channels and T-channel-dependent currents [61].

##### TRP channels

Altered expression and function of several thermo- and mechanosensitive Transient Receptor Potential (TRP) channels, namely, vanilloid 1 (TRPV1), Ankyrin-repeat 1 (TRPA1), and melastatin 8 (TRPM8) [43, 46, 47, 90, 91], and TRPA1- and TRPV4-dependent oxidative stress have also been reported in CIPN [47, 92–95].

In mice, oxaliplatin-induced rapid-onset cold hypersensitivity is ameliorated by TRPA1 blockade [49, 96]. Oxaliplatin stimulates TRPA1 but does no effect on TRPM8 or TRPV1 channels either in vivo or in vitro. These responses are caused by the oxaliplatin metabolite—oxalate. In human TRPA1-expressing cells, oxaliplatin and oxalate cause TRPA1 sensitization to reactive oxygen species (ROS) by inhibiting prolyl hydroxylases, which is consistent with the observed cold hypersensitivity. Hence, it is thought that oxaliplatin-induced acute cold hypersensitivity is caused by TRPA1 sensitization to ROS via enzyme inhibition, which enables TRPA1 to convert ROS signals into cold sensitivity [47, 96].

Oxaliplatin-induced TRPA1 activation occurs via a glutathione-sensitive mechanism [94, 96]. In a study by Miyake and colleagues, a high concentration of oxaliplatin increased intracellular calcium concentration in human (h) TRPA1-expressing HEK293 cells. Oxaliplatin also induced the rapid generation of hydrogen peroxide and evoked ROS-mediated cysteine oxidation-dependent hTRPA1 activation. The observed calcium influx was prevented in the presence of glutathione. In contrast to this, a lower concentration of oxaliplatin (100 μM) did not increase the intracellular calcium concentration but caused the cold-induced cysteine oxidation-dependent opening of human TRPA1 channels. These concentration-dependent biochemical mechanisms of the observed phenomena were different [96].

Paclitaxel activates and sensitizes the function of TRPV1 and TRPV1 antagonists induced analgesia in paclitaxel-related CIPN [17]. TRPA1 and TRPM8 channels have also been implicated in paclitaxel-induced neurotoxicity and CIPN-related pain behavior in mice [97]. A recent study also confirmed a role of TRPV4 channels in CIPN caused by paclitaxel. In this study [93], the administration of paclitaxel resulted in strong IP_3_-mediated calcium signals amplified by calcium entry through TRPV4 channels. Blocking calcium influx through TRPV4 channels reduced cell death in cultured dorsal root ganglia neurons, and the pretreatment of mice with the TRPV4 inhibitor (HC067047) prior to paclitaxel injection prevented both the neurotoxic effects of paclitaxel on electrophysiology and behavior [93].

The exposure of isolated sensory neurons to vinca alkaloids produces an inward sodium current that depolarizes these neurons, resulting in neuronal firing. These neuronal effects are regulated by TRPA1 channels, and hypersensitivity to painful stimuli in response to vinca alkaloid administration is reduced in TrpA1 mutant flies and mice [98].

TRPA1 channels are also involved in bortezomib-induced neurotoxicity, and the selective blockade of these channels by Phα1β (a peptide from the venom of the armed spider *Phoneutria nigriventer*) and CTK 01512-2 (a recombinant form of the peptide) reduces the TRPA1-dependent neuropathic pain-like responses induced by bortezomib [99].

#### Activation of the immune system and neuroinflammation

Antitumor drugs modulate the immune system, and this constitutes one of the most important mechanisms underlying tumor cell destruction. Simultaneously, the activation of the immune system, the recruitment of immune cells and neuroinflammation are regarded as a potential significant contributors to the development of CIPN [17, 100–104].

An increase in the peripheral levels of proinflammatory cytokines, changes in immune signaling pathways and a strong correlation between inflammation and peripheral neuropathy caused by chemotherapy have been observed in numerous studies [102, 105, 106].

It was recently shown that vincristine dysregulates genes associated with immunological processes, whereas oxaliplatin causes the dysregulation of genes associated with neuronal function. Cisplatin influences the expression of genes implicated in both inflammatory and neuropathic pathologies [102]. The infiltration of leukocytes into the nervous system of animals with CIPN symptoms and peripheral and central glial cell activation have also been observed. These alterations are dependent on the chemotherapeutic drug used, its dose, the treatment schedule, and the therapy duration. There is, however, still limited evidence for such phenomena from human studies [105].

##### Cytokines and chemokines

Increased production and release of cytokines [e.g., interleukins (IL): IL-1β, IL-6, IL-8, tumor necrosis factor α (TNFα), and interferon γ (IFN-γ)] and chemokines (e.g., CCL2, and CXCL12, CCL11, CCL3, and CCL4) and decreased expression of anti-inflammatory cytokines (IL-10 and IL-4) are observed after the administration of several chemotherapeutics including paclitaxel, cisplatin and vincristine. IL-1, TNFα, and IL-6 not only cause axonal damage but also facilitate neuron-immune communication and increase the release of bradykinin, serotonin, and histamine. These mediators are able to augment proinflammatory processes and act as sensitizers for nociceptors, thus playing a crucial role in the progression of CIPN. Increased concentrations of cytokines and chemokines in the dorsal root ganglia and spinal cord induce alterations in the Schwann cells of peripheral axons, satellite cells in the dorsal root ganglia, and astrocytes in the spinal cord, which all contribute to CIPN development [102, 106–111].

Recently, using models of CIPN induced by paclitaxel and oxaliplatin, it was shown that the IL-8 signaling pathway is involved in neuroinflammation that results in a progressive neural sensitization [108]. The levels of interleukin IL-6 and IL-6 receptors in women after breast cancer treatment have also been investigated, and these studies revealed that the IL-6 signaling pathway may be an important biological mechanism associated with the persistence of the painful symptoms of CIPN, which may have potential implications for the management of CIPN symptoms [112].

Oxaliplatin administration increases the mRNA levels of proinflammatory cytokines and chemokines, and this effect is strongly correlated with the development of mechanical hypersensitivity observed in rats. Oxaliplatin-induced pain is accompanied by the upregulation of PI3K-mTOR, enhanced mTOR-mediated signals and increased ERK phosphorylation. Taken together, these findings suggest that the mechanism of the development of oxaliplatin-induced neuropathy resembles that of inflammatory pain [113].

Crosstalk between the nervous system and the immune system during chemotherapy is well known to occur, and the upregulation of chemokines is a key phenomenon that modulates this effect. Chemokines, which were originally identified as regulators of peripheral immune cell trafficking, are expressed on neurons and glial cells in the central nervous system. As such, they are regarded as important contributors to pain signaling in CIPN. The expression of chemokines and their receptors, (e.g., CX3CL1/CX3CR1, CCL2/CCR2, CXCL1/CXCR2, CXCL12/CXCR4 and CCL3/CCR5) is altered in CIPN [114, 115].

The C–C motif chemokine ligand 2 (CCL2, also known as monocyte chemoattractant protein 1, MCP1) [2] and its receptor CCR2 are increased in the small dorsal root ganglion neurons of paclitaxel-treated rats with CIPN symptoms. CCR2 gene knockdown or CCR2 blockade reduces neuropathic pain in mice [17, 116].

In addition to increasing the level of CCL2, paclitaxel also increases the level of CCL3 in the lumbar dorsal root ganglion [109].

Increased levels of CCL2 and CCR2 in the dorsal root ganglia have also been observed to be accompanied by mechanical hypersensitivity in oxaliplatin-treated rats [117].

Using a mouse model of vincristine-induced CIPN, Montague and colleagues demonstrated that CCL2/CCR2 signaling plays a crucial role in the development of allodynia in CX3CR1-deficient mice. They suggested that this effect might be due to an interaction between CX3CR1 and CCR2 receptors in monocytes [118].

Vincristine and paclitaxel upregulate C-X-C motif chemokine ligand 12 (CXCL12) in the dorsal horn ganglia. This chemokine is a ligand of CXCR4 (CD184, C-X-C chemokine receptor type 4). CXCL12 can be released from the central terminals of dorsal root ganglion neurons into spinal cord dorsal horn neurons and functions as an attractant for T-lymphocytes and monocytes. The activation of CXCR4 increases the intracellular calcium concentration and stimulates the chemotaxis of immune cells to the site of inflammation [17].

##### Immunological ligands and receptors

Toll-like receptors (TLR) are transmembrane proteins that function as sensors of a diverse range of endogenous and exogenous substances that are potentially harmful to the body. They are widely expressed on immune cells, enterocytes, sensory neurons and glial cells. When activated, they modulate inflammatory responses in body tissues to detect various pathogens. TLR4 is specialized to detect bacterial pathogens, and TLR3 detects viral pathogens. TLR4 is also activated in many neuropathic pain states [119], including those associated with CIPN caused by paclitaxel [120–125], oxaliplatin [126, 127], cisplatin [6, 128] and vincristine [129]. TLR4 activation by chemotherapeutics is responsible for the increase in proinflammatory cytokine expression in the peripheral and central nervous systems. TLR4 signals that promote the expression of MCP-1 are increased in the dorsal root ganglia of rats with paclitaxel-induced hyperalgesia, and this effect can be prevented by cotreating the animals with TLR4 antagonists during chemotherapy [116].

Similarly, mice with genetic TLR4 or TLR3 knockout do not develop hyperalgesia after treatment with cisplatin [17, 110].

Sex-dependent differences in pain sensitivity were shown recently in paclitaxel-treated rats with CIPN symptoms [130], and it has been demonstrated that the lipopolysaccharide-induced activation of spinal TLR4 mediates mechanical allodynia in male mice but not in female mice. This receptor also plays a sex-specific role in inflammatory and neuropathic pain in male mice, and this sex-specific response is limited to the spinal cord [131].

The cellular and molecular mechanisms of TLR9 signaling in CIPN caused by paclitaxel were investigated using Tlr9-deficient mice of both sexes. It was found that in male mice but not in female mice, a Tlr9 mutation attenuated the development of neuropathic pain caused by paclitaxel. Moreover, a TLR9 antagonist injected intraplantarly alleviated paclitaxel-induced mechanical allodynia only in male wild-type mice. Taken together, this study shows the involvement of sex-dimorphic TLR9 signaling (in both the spinal cord and the periphery) in CIPN and suggests that TLR9 signaling pathway promotes CIPN in male mice. It was postulated that the activation of TLR9 in macrophages in male mice results in the release of proinflammatory cytokines and chemokines and that this is the basis for CIPN-related mechanical allodynia in male mice [120].

##### Immune cells

*T-cells *Paclitaxel-induced mechanical allodynia is prolonged in T-cell-deficient (Rag1−/−) mice compared to their wild-type littermates, and it was demonstrated that CD8 + T-cells in the dorsal root ganglia are critical for recovery from CIPN [110, 132]. Similar results have been shown for cisplatin [133]. T cells are also involved in the preventive effects of histone deacetylase six inhibitors on cisplatin-induced mechanical allodynia and mitochondrial deficits in dorsal root ganglion neurons [134].

On the other hand, in male C57BL/6 J mice treated with paclitaxel or oxaliplatin, significant mechanical allodynia is accompanied by increased circulating CD4 + and CD8 + T-cells [109].

*Macrophages * The intestinal mucosa is the largest immune organ and contains an abundance of macrophages, which are key cells for homeostasis maintenance. Macrophages regulate the development of behavioral hypersensitivity and the loss of distal epidermal nerve fibers and hence play an important role in paclitaxel-related neuropathy [116, 135].

It was recently postulated that the gut microbiota may influence CIPN through macrophages [110], and after chemotherapy, the number of CD11b + CD45^hi^ cells, which are presumably macrophages, is significantly lower in the dorsal root ganglia of mice that receive water containing antibiotics than those receiving water alone [136].

In rats, intravenous immunoglobulins are effective as a therapeutic option, reducing macrophage infiltration and the severity of bortezomib-induced CIPN [103].

*Astrocytes *As the most abundant cells in the CNS, astrocytes participate in diverse functional processes, including brain metabolism and neuronal transmission. The activation of astrocytes and the release of modulators from these stimulated cells have been implicated in neuropathic pain of various origins [110], including paclitaxel-induced CIPN [107, 109].

*Microglia *The role of spinal microglia in neuropathic pain syndromes is complex [137] and, at least for CIPN, is not completely understood. Investigations of the role of microglia in CIPN have presented opposing findings. Some researchers gave reported increased microglial activation in response to vincristine and paclitaxel administration [138–141]. On the other hand, there are also data that suggest that microglia do not play a significant role in CIPN, at least when compared to the pivotal role of astrocytes in this clinical entity [142].

It should be emphasized that microglia, as immune cells, are an abundant source of pain-associated substances, including IL-1β, IL-6, and TNFα. Minocycline, an inhibitor of macrophages, monocytes and microglia, as well as a matrix metalloproteinase-9 (MMP-9) inhibitor, attenuates mechanical hyperalgesia induced by oxaliplatin and paclitaxel [17, 110].

In cisplatin-treated mice that show symptoms of mechanical allodynia and sensory deficits, the activation of microglia, but not astrocytes, in the spinal cord is observed. This is accompanied by increased mRNA levels of inflammatory state-related molecules (IL-1β, IL-6, TNFα, and inducible nitric oxide synthase). Minocycline alleviates cisplatin-induced behavioral signs of neuropathy in this mouse model [143].

A large number of studies have focused on the connection of the gut microbiota to chemotherapy-induced diarrhoea or mucositis [110], but there is also recent evidence for the involvement of the gut microbiome and impaired microglia function in the development of CIPN [104, 136, 144]. The microbiota is critical for controlling microglial maturation and function, and the treatment of adult mice with antibiotics results in impaired maturation and innate immune functionality of these immune cells in the brain. It has been hypothesized that the gut microbiome connects the microbiome-gut-brain and neuroimmune-endocrine axes, and this network might influence key components involved in CIPN.

Recently, a potential role of the gut microbiome in the development of CIPN caused by paclitaxel was suggested [138]. Spinal microgliosis is also involved in paclitaxel-induced pain and a link between the gut microbiota, microglial activation and neuroinflammation in CIPN has been reported [144]. In this study, paclitaxel decreased the amount of beneficial bacteria such as *Akkermansia muciniphila* OTU, which was previously shown to promote the integrity of the epithelial cell layer and improve barrier function [144]. It was then hypothesized that chemotherapy causes barrier dysfunction, resulting in increased systemic exposure to bacterial products and metabolites, which in turn promotes systemic inflammation and pain. Also, oxaliplatin-induced mechanical hyperalgesia is reduced in germ-free mice and in mice pretreated with antibiotics, which further suggests that the gut microbiota promotes the development of oxaliplatin-induced mechanical hypersensitivity and neuroinflammation. These effects appear to be mediated, in part, by TLR4 expressed on macrophages [136].

*Schwann cells *Schwann cells are glial cells in the peripheral nervous system that form a thin myelin sheet around the axons of motor and sensory neurons, thus enabling saltatory conduction of action potentials. Furthermore, they are a source of vasoactive mediators and play an important role in the recruitment of immune cells from the vasculature. Schwann cells have been shown to express various TLRs and play a key role in neuroinflammation resulting from the administration of antitumor drugs [29, 110]. Recently, Imai and colleagues investigated the effects of antitumor drugs on Schwann cells. They showed that the exposure of primary cultured rat Schwann cells to paclitaxel, cisplatin, or oxaliplatin at concentrations lower than those required to induce neurotoxicity in cultured rat dorsal root ganglion neurons induced cytotoxicity and reduced myelin basic protein expression. These chemotherapeutic drugs disrupted myelin formation by Schwann cells. Platinum derivatives induced mitochondrial dysfunction in cultured Schwann cells, while paclitaxel led to the dedifferentiation of Schwann cells into an immature state characterized by increased expression of p75 and galectin-3. In line with this, in the mouse sciatic nerve, repeated injection of paclitaxel increased the expression of p75 and galectin-3 in Schwann cells [145].

##### Central nervous system structures and neurotransmitters

In the course of CIPN structural damage to the peripheral nervous system induces abnormal central nervous system processing. The involvement of the brain and spinal cord in CIPN development has become a subject of extensive studies but at present the impact of CIPN on brain structures and levels of central nervous system neurotransmitters is still understudied.

To elucidate the role of central nervous system neurons in altered behavioral sensitivity seen during chronic pain conditions Samineni and colleagues [146] studied spontaneous and thermally-evoked firing patterns of ventrolateral periaqueductal gray neurons in rats treated with paclitaxel. In this research, ventrolateral periaqueductal gray neurons displayed increased neuronal activity and changes in thermal pain-evoked neuronal activity. This involved elevated levels of spontaneous firing and increased responsiveness to non-noxious stimuli (allodynia) as well as noxious thermal stimuli (hyperalgesia) as compared to controls. Furthermore, after paclitaxel treatment only excitatory neuronal responses were observed to both non-noxious and noxious thermal stimuli. Systemic administration of gabapentin induced significant dose-dependent decreases in the elevated spontaneous and thermally-evoked ventrolateral periaqueductal gray neuronal firing to both non-noxious and noxious thermal stimuli in paclitaxel-treated rats but not in naïve rats. These results showed a strong correlation between behavioral hypersensitivity to thermal stimuli and increased firing of ventrolateral periaqueductal gray neurons in allodynia and hyperalgesia that occur after paclitaxel administration.

The reorganization of neural circuits in the brain in CIPN have been also observed by Ferris and colleagues [147]. They conducted an imaging study to evaluate the impact of paclitaxel treatment (8 days) on brain neural circuitry in rats. Paclitaxel-treated rats were more sensitive to a cold stimulus compared to controls. Brain areas involved in the emotional and motivational responses to chronic pain were also impacted by paclitaxel treatment. Affected brain regions included the prefrontal cortex, amygdala, hippocampus, hypothalamus and the striatum/nucleus accumbens. This study confirmed central nervous system effects of chemotherapy and showed that neuropathic pain is modulated by emotion and motivation, and influences activity of the periaqueductal gray and brainstem to modulate pain perception.

The central changes observed during paclitaxel-induced CIPN are likely to be caused by the neurotoxic effects triggered by this antitumor drug at the periphery because paclitaxel has a very low ability to cross the blood–brain barrier [148].

CIPN-related alterations are also observed at the spinal cord level and paclitaxel-injected animals showed increased spontaneous activity of wide-dynamic range neurons and decreased expression of glutamate transporters in the lumbar spinal dorsal horn. These results suggest a state of increased excitability that develops in spinal pain-signaling neurons as a consequence of decreased glutamate clearance. These changes in dorsal horn contribute to the hyperresponsiveness to sensory stimuli seen in animals treated with paclitaxel and may play a role in pain seen in patients on paclitaxel therapy [149].

Reorganization of some brain areas, including the frontal lobe, insular cortex, somatosensory cortex, thalamus, periaqueductal gray and precuneus has been demonstrated in CIPN. A study using fMRI explored central pain processing in myeloma patients with CIPN. This study showed that painful stimuli delivered to neuropathy-affected and symptom-free sites in patients suffering from CIPN evoked differential activation of distinct cortical regions [150].

An association between cerebral resting-state perfusion and CIPN symptoms was demonstrated in patients with breast cancer and CIPN symptoms. These results suggest that gray matter density decrease and associated perfusion decrease may interfere with CIPN-symptoms and patient symptom perception. Individuals showing gray matter density decrease may report less severe symptoms of CIPN, while showing less CIPN symptom-related perfusion change [151].

Altered levels of several neurotransmitters, such as catecholamines, histamine, serotonin, glutamate and γ-aminobutyric acid (GABA), are associated with CIPN caused by vincristine [152, 153], paclitaxel [154], oxaliplatin [155–157] and bortezomib [158].

The analgesic effect mediated by monoamines: serotonin and noradrenaline, is due to the blockade of nociceptive transmission at the spinal dorsal horn. This activity is independent of their antidepressant effect. Antidepressants that act at serotonin and noradrenaline reuptake are clinically relevant in CIPN treatment [19].

Using an animal model of paclitaxel-induced CIPN, increased activity of serotoninergic neurons of the rostroventromedial medulla has been demonstrated in rats. The immunohistochemical analysis of serotonin showed increased expression at the superficial dorsal horn (laminae I–II) and higher levels of 5-HT_3_ receptors in the superficial dorsal horn (laminae I–II) of paclitaxel-injected animals. The intrathecal administration of the 5-HT_3_ receptor antagonist (ondansetron) reversed mechanical and cold hypersensitivity of paclitaxel-treated rats. These results indicate that CIPN is associated with increased recruitment of descending serotonergic modulation from the rostroventromedial medulla which affects the spinal serotoninergic system and might account for pain hypersensitivity mediated by spinal 5-HT_3_ receptors [159].

Animal models of neuropathic pain revealed that noradrenaline is a neurotransmitter that plays an important role in the inhibition of this chronic pain type. The increase of noradrenaline in the spinal cord by reuptake inhibition directly attenuates neuropathic pain through *α*_2_-adrenergic receptors. Moreover, noradrenaline acts within the locus coeruleus and improves the function of the impaired descending noradrenergic inhibitory system. Serotonin and dopamine may reinforce the noradrenergic effects to inhibit neuropathic pain [160].

The role of descending noradrenergic modulation of spinal nociceptive transmission in CIPN was studied in paclitaxel-treated rats [26]. It has been shown that in the paclitaxel-induced CIPN rat model, the descending noradrenaline-mediated inhibition affecting nociceptive transmission at the spinal cord is potentiated. It has been suggested that the enhanced noradrenergic inhibition during CIPN may represent an adaptive mechanism of the descending noradrenergic pain control system to the increased peripheral nociceptive input. Pharmacological stimulation of *α*_2_-adrenergic receptors at the spinal cord level using noradrenergic drugs (e.g., reboxetine, clonidine) may, therefore, represent a promising therapeutic opportunity to treat CIPN-related pain [26].

Glutamate transporters, such as glutamate transporter 1 (GLT-1) and glutamate aspartate transporter (GLAST, EAAT1), are abundantly expressed in astrocytes, where they act to remove synaptically released glutamate from the synaptic cleft [161]. These transport proteins have been shown to be downregulated in activated astrocytes, and this results in impaired functioning of these proteins, leading to neuronal hyperexcitability. Importantly, the downregulation of both GLT-1 and GLAST has been observed in paclitaxel-treated [149, 162, 163], oxaliplatin-treated [164] and bortezomib-treated neuropathic rats [165]. In our recent study [166], a single dose and repeated doses of ceftriaxone, a beta-lactam antibiotic that acts by upregulating GLT-1 expression, thus increasing glutamate reuptake in the CNS [167–169], was tested for its ability to attenuate early-phase and late-phase tactile allodynia and cold hyperalgesia in oxaliplatin-treated mice. The repeated intraperitoneal administration of 200 mg/kg ceftriaxone prevented the development of late-phase tactile allodynia, but ceftriaxone showed no antiallodynic properties in the cold plate test. These results confirmed previous findings showing that ceftriaxone (through increased GLT-1 expression) affected mechanical rather than thermal (cold) allodynia, which suggests that the biological functions of GLT-1 are more associated with the regulation of the mechanical nociceptive threshold than the cold nociceptive threshold [170].

In addition to decreased astrocytic glutamate reuptake due to GLT-1 and GLAST hypofunction, increased synaptic γ-aminobutyric acid (GABA) reuptake resulting from the enhanced GAT-1 function should be considered as a phenomenon implicated in CIPN development. The evidence for this is mainly based on the observed significant antiallodynic and antihyperalgesic effects of GAT-1 inhibitors in rodent models of CIPN. Decreased GABAergic neurotransmission due to excessive IL-17 levels in the spinal cord has been linked to CIPN caused by paclitaxel [171], and the inhibition of GABA reuptake with by NO-711, a selective GABA transporter isoform 1 (GAT-1) inhibitor [172], prevents the development of thermal hyperalgesia and allodynia in paclitaxel-treated mice. NO-711 is also therapeutically effective for pre-existing CIPN [173]. Also, GABAergic precursor cell transplants reverse paclitaxel-induced mechanical hypersensitivity [174].

In paclitaxel-treated animals, the enhancement of GAT-1 protein expression, decreased GAT-3 expression, increased global GABA uptake, as well as suppression of GABAergic tonic inhibition in the spinal dorsal horn are also noted. Paclitaxel-induced neuropathic pain was significantly attenuated by the intrathecal injection of a GAT-1 inhibitor but not a GAT-3 transporter inhibitor. These findings confirm that targeting GAT-1 transporters for reversing disinhibition in the spinal dorsal horn may be a useful approach for treating paclitaxel-induced neuropathic pain [175].

In oxaliplatin-treated rats, the elevation of IL-1β, IL-6, and TNFα levels in the periaqueductal gray matter and the upregulation of IL-1β, IL-6, and TNFα receptor expression in the plasma membrane in the periaqueductal gray of oxaliplatin-treated rats coincides with decreased GABAergic neurotransmission. The behavioral symptoms noted in these animals include tactile and cold allodynia. The blockade of proinflammatory cytokine receptors results in restored GABA function and the attenuation of mechanical and cold hypersensitivity [176].

The effect of tiagabine, a highly selective GAT-1 inhibitor, on oxaliplatin-induced neuropathic pain in mice was assessed [177]. The intraperitoneal administration of tiagabine at doses of 4 mg/kg and 8 mg/kg attenuated tactile allodynia in the von Frey test both 3 h (acute phase of CIPN) and 7 days (late phase of CIPN) after oxaliplatin injection. Tiagabine was not effective in reducing cold hyperalgesia in either the acute phase or the late phase.

The effect of non-GAT-1 inhibitors on the behavioral symptoms of CIPN is less known; however recent studies have shown that preferential inhibitors of mouse GAT3/4 might attenuate tactile allodynia but not cold allodynia in oxaliplatin-treated mice [178].

## Concluding remarks

Since the survival rate of patients treated with antitumor agents is increasing, CIPN is considered to be a significant cause of decreased quality of life among cancer survivors. The number of analgesic drugs that effectively relieve pain symptoms and are used as an interventional treatment for pre-existing CIPN-related pain is very limited, and the efficacy of these drugs in CIPN is significantly lower than that in other neuropathic pain types. Despite our growing knowledge regarding the mechanisms underlying CIPN, this clinical entity is still not preventable. Currently, animal models of CIPN are widely used to investigate CIPN pathophysiology and to develop potential novel therapies for this debilitating condition. These preclinical models aim to reflect the impairments observed in CIPN in humans and they provide a deeper insight into the mechanisms underlying the initiation and maintenance of CIPN.

On the other hand, the lack of translational progress in pain studies has been observed, recently. Basic science knowledge obtained using both in vivo and in vitro models has not resulted in a dynamic development of novel drugs for CIPN and even a broader use of genetically-manipulated animals to study CIPN or assays that more closely resemble clinical picture of CIPN-related pain states has not increased the effectiveness of drug discovery and development of analgesics for CIPN. Therefore, a careful re-examination of animal models of CIPN-related pain seems an urgent need.

Pain is a complex phenomenon which is affected by a wide range of modulatory factors, including sex, genotype and social communication, and all of them must be considered when translating results obtained in animal model to humans [179–182]. In addition to genetic and neurochemical differences between rodents and humans, there are also neuroanatomical differences in pain pathways between rodents and humans (i.e., rodent and primate dorsal horns, the forebrain) and this might be crucial for the observed distinct pain sensation. Hence, a greater emphasis on studies of pain in humans is strongly recommended [179].

Importantly, previous pain studies were focused on the sensory dimension of pain with almost no analysis of the affective component of pain, and empathy-like responses in experimental animals were, therefore, neglected [183]. The affective aspect of pain is still not well-addressed, so there is a strong need to study the affective component of pain in animal models [182].

Several other factors should be also considered at the preclinical phase as potential factors leading to translational failure. Firstly, animal models might not be the best representation of the clinical situation and it is of key importance to use only the most effective and reliable animal models that mimic the clinical situation as close as possible (construct validity). Scientists need to be aware of the existence of various available models, and how they differ in characteristics and efficacy in causing CIPN. In this context, it seems that the available animal CIPN models are manifestations of gain in sensory functions which measure mainly allodynia, hyperalgesia and neurophysiological alterations in nerve function, whereas many patients, at least in the chronic phase of CIPN also report other symptoms, such as numbness, tingling and ongoing pain. This may compromise the clinical relevance of these animal models for chronic CIPN studies [181, 184]. Therefore, the use of animal models that replicate all symptoms observed in humans is strongly demanded but is still a very challenging endeavor as some symptoms occur spontaneously and are very difficult to replicate in animals. At present, measures of ongoing pain in rodents is an emerging phenomenon.

Another key issue regarding the clinical relevance of animal models is that the majority of them utilize cancer-free animals, whereas in the clinical situation most CIPN patient have or experienced previously cancer. Cancer may significantly confound the effectiveness (both: pharmacokinetic and pharmacodynamics properties) of drugs used for CIPN treatment [181, 184].

Also, doses and mode of deliveries of the anti-tumor drugs may not reflect the clinical situation, and many preclinical studies do not seem to be fully randomized and blinded.

Polyneuropathy characteristics is also important and there are some serious concerns about the clinical relevance of the time courses frequently studied in animals. Acute CIPN affects patients within the first 6 months and chronic CIPN has been observed in patients approximately 2 years after treatment. Therefore, the short duration of the majority of animal models of CIPN likely regard the acute phase only [181].

Finally, most studies use only male animals and evoke limb withdrawal to mechanical stimuli [180, 181]. In the clinic, many CIPN-inducing drugs are frequently used to treat female cancer patients (e.g., ovarian or breast cancers), thus the use of both sexes of animals in the modeling of CIPN is warranted and will likely improve the validity of animal models [26, 181].

Taken together, it is important to realize that at present no animal model represents the full clinical situation perfectly because of interspecies differences and inevitable differences between the conditions created in animals and those observed in human CIPN. Research findings, therefore, need to be confirmed in multiple animal models as comparable results in multiple animal models of CIPN would increase our confidence in the results and their applicability for the clinical situation.

Since many of the mechanisms that underlie CIPN overlap, they may reinforce each other. Hence, combination therapies seem to be potentially useful and valuable approaches to prevent CIPN development or treat its symptoms. Unfortunately, at present, the number of clinical trials focused on testing combination drug therapies for neuropathic pain is scarce, and there are no clear algorithms describing how to use combined drugs in neuropathic patients.

Importantly, the patient-reported severity of CIPN symptoms are more valuable than the assessments provided by clinicians. One should also realize that comorbidities present in patient populations in addition to cancer itself are likely to influence nociception and CIPN-related neuropathic pain pathophysiology. Therefore, a more effective treatment for CIPN will require close cooperation between oncologists, neurologists, psychiatrists and specialists from other fields of medicine to ensure that CIPN patients are effectively cured and monitored.

A better understanding of the mechanisms of CIPN is important for the identification of novel preventative therapies. Deeper insight into mechanisms underlying axonal degeneration in CIPN is, therefore, an urgent need. Since degenerative processes within the peripheral nervous system are only a part of CIPN, we also need to understand the role of central nervous system changes (neuroplasticity and the central sensitization of neuropathic pain), neuromodulators, neurotransmitters and receptors implicated in the phenomena that induce CIPN [185].
